# Efficient delivery of small interfering RNA for inhibition of IL-12p40 expression *in vivo*

**DOI:** 10.1186/1476-9255-1-4

**Published:** 2004-10-01

**Authors:** Marion A Flynn, David G Casey, Stephen M Todryk, Bernard P Mahon

**Affiliations:** 1 Institute of Immunology, National University of Ireland, Maynooth, Co. Kildare, Ireland

## Abstract

**Background:**

RNA interference is an evolutionary conserved immune response mechanism that can be used as a tool to provide novel insights into gene function and structure. The ability to efficiently deliver small interfering RNA to modulate gene expression *in vivo *may provide new therapeutic approaches to currently intractable diseases.

**Methods:**

*In vitro*, siRNA targeting IL-12p40 was delivered to the murine macrophage cell line (J774A.1) encapsulated in a liposome with an IL-12 inducing agent (LPS/IFN-γ) over a number of time points. Controls included a variety of non-target specific siRNA reagents. Supernatants were analyzed for cytokine production while the cells were removed for mRNA profiling.

*In vivo*, siRNA-targeting IL-12p40 was delivered to the murine peritoneal cavity in a therapeutic fashion, after endotoxin (LPS) challenge. Cells from the peritoneal cavity were removed by lavage and analyzed by flow cytometry. Levels of IL-12 present in lavage and in serum were also examined by ELISA.

**Results:**

In this report, we show that IL-12p40 siRNA can specifically silence macrophage expression of IL-12p40 mRNA and IL-12p70 protein *in vitro*. We extend this finding to demonstrate that delivery of liposome encapsulated siRNA targeting IL-12p40 to the murine peritoneal cavity can modulate an inflammatory stimulus *in vivo*. Furthermore, specific siRNA can be used therapeutically after endotoxin challenge to reduce both the local and systemic inflammatory response. Thus, the delivery of siRNA can be used to elicit specific non-permanent inhibition of endogenous protein expression.

**Conclusion:**

*In vitro *silencing of IL-12p40 using siRNA at selected doses leads to specific knockdown of IL-12p70 protein production without inducing type I interferons. Furthermore, siRNA targeting murine IL-12p40 can be used therapeutically to counter an inflammatory response *in vivo*.

## Background

RNA interference (RNAi) is an evolutionary conserved sequence-specific RNA silencing mechanism found as an anti-viral response in invertebrates, plants and mammalian cells [1]. Although the mechanism of silencing is not completely understood, the basic premise of RNAi rests on the ability of double stranded RNA (dsRNA) to specifically degrade homologous messenger RNA (mRNA). The RNAi pathway is triggered in mammalian cells by the presence of dsRNA or in the presence of short 19–22nt dsRNA fragments termed small interfering RNA molecules (siRNA). siRNA molecules activate an RNA-induced silencing complex (RISC) that unwinds the siRNA duplex [2]. The specificity of locus degradation is guided by the antisense strand of the unwound siRNA, followed by sense strand siRNA binding to the complementary mRNA site for cleavage by RISC. The cleavage of the sense strand siRNA and target mRNA results in the self-amplifying production of new siRNA intermediaries that continue mRNA target degradation in an ATP dependent manner [3,4]. This phenomenon means that low doses of siRNA can be more effective than antisense therapy. Furthermore, this approach is preferable to gene and antisense based therapies, in that siRNA is non-heritable and does not require adenoviral vectors, which limit the effectiveness and acceptability for use in children.

RNAi can be exploited as a tool to provide novel insights into gene function and structure. The capacity to efficiently deliver siRNA to modulate gene expression *in vivo *may provide new therapeutic approaches to currently intractable diseases. Like other new genetic technologies, siRNA gene suppression faces several methodological limitations *in vivo. *Foremost among these are the efficient delivery of siRNA to target cells [5,6], non-specific effects of putative control duplexes [7-9] and the potential therapeutic problems of viral expression vectors [10]. One approach to overcoming these obstacles is to deliver non-heritable siRNA duplexes in a model system and monitor the influence upon experimentally induced inflammation. This approach would provide a method that allows the rapid screening of what have been termed "druggable" targets [11].

Interleukin-12 (IL-12p70) is a cytokine with a well-characterized pro-inflammatory function [12] that has been suggested as a target for therapeutic intervention [13-15]. Bioactive IL-12p70 is a heterodimer formed by a heavy chain (p40) and a light chain subunit (p35), encoded by two separate genes whose expression is independently regulated at the transcriptional level [16]. The p35 sub-unit is constitutively expressed at low levels in most cell types but is up regulated during cell activation. In contrast, the IL-12p40 gene is under tight transcriptional control only expressed in macrophages or other APC following activation by microbial products [17]. Production of IL-12p70 is enhanced by IFN-γ via the IFN consensus sequence binding protein [18] but reduced by IL-10 [19].

IL-12p70 has pleiotropic effects on target cells but the major role is as a pro-inflammatory cytokine in cell mediated immunity against microbial insult. In particular IL-12p70 acts upon T and NK cells to increases cytokine production, proliferation, and cytotoxicity, functions that become evident several hours after exposure to infections agents [19]. The IFN-γ subsequently produced, potentiates antigen presentation functions important in clearing infectious agents. These functions include increased co-stimulatory molecule expression, phagocytosis, and production of reactive oxygen and nitrogen intermediates [19,20]. However, IL-12p70 is not always protective or beneficial, indeed a variety of pathological conditions, including sepsis, are associated with IL-12 driven pathology [21,22]. In addition to the well-characterized role of IL-12p70, it is now known that the IL-12p40 subunit is also biologically active. This subunit may act to antagonize the heterodimer function [23], or may have a broader direct role, less dependent on IL-12p70 [24,25].

In order to explore the therapeutic feasibility of RNA interference, we used siRNA to specifically ablate IL-12p40 expression *in vitro *and *in vivo*. This approach extends the power of RNA interference to gene expression studies in live animals without the use of genetic engineering, plasmid DNA reporter systems [2,26] retroviral [27,28] or lentiviral siRNA expression vectors [29] and opens the way for exploring the use of siRNA in humans to treat disease. Our results provide a description of siRNA mediated suppression of an endogenous immune gene *in vivo *and describe a novel therapeutic and research approach for gene specific inhibition of an important cellular and immunological response.

## Materials and Methods

### Mice & Cell Lines

Female BALB/c mice (Harlan Limited, Bicester, UK) and IL-12p40 gene-disrupted mice (IL12p40^-/-^) (Jackson Laboratories, Bar Harbor, Maine) were maintained under the guidelines of the Irish Department of Health and the local bioethics committee. All mice were 12–14 weeks old at the initiation of experiments and sacrificed on completion. The murine macrophage cell line (J774A.1) was used to investigate silencing of IL-12p40 cytokine gene expression.

### Preparation of siRNA

siRNA oligonucleotides with the following sense and antisense sequences were designed from the GenBank repository: accession number; NM_008352, *Mus musculus *interleukin 12b (IL12b), mRNA. IL-12p40 siRNA 5'-C CUC ACC UGU GAC ACG CCU dTdT-3' (sense) and 3'-dTdT G GAG UGG ACA CUG UGC GGA-5' (antisense); Mutant siRNA 5'-C CUC ACC UUC GAC ACG CCU dTdT-3' (sense) and 3'-dTdTG GAG UGG AAG CUG UGC GGA-5' (antisense); GFPsiRNA 5'-GGC UAC GUC CAG GAG CGC ACC dTdT-3' (sense) and 3'-dTdT CCG AUG CAG GUC CUC GCG UGG-5' (antisense). The antisense of the IL-12p40 siRNA duplex (As.RNA) was also used as a control for *in vivo *experiments. Each complementary RNA strand was deprotected according to manufacturer's instructions. For the production of the IL-12p40 siRNA duplex, sense and antisense siRNA strands were mixed in equimolar ratios and treated by heating to 95°C for 1 min followed by annealing at 37°C for 1 h and allowed to cool slowly overnight to room temperature. All siRNA oligonucleotides were synthesized commercially (Dharmacon, Lafayette, CO) using 2'ACE protection chemistry.

### *In vitro *siRNA interference

Semi-confluent J774A.1 cells were cultured at 1 × 10^5 ^cells/ml in antibiotic free, 8% (v/v) endotoxin-low fetal-calf serum RPMI (Gibco-Invitrogen, Paisley, UK) containing L-glutamine (Sigma, Poole, UK) 12–16 h before transfection. For siRNA transfections 3 μl of a 20 μM siRNA duplex (target or control) solution was mixed with 47 μl of Opti-mem (Gibco-Invitrogen). In a second tube 3 μl of oligofectamine (Gibco-Invitrogen) was mixed with 12 μl of Opti-mem and incubated at room temperature for 15 min. Solutions were combined for 40 min and brought to a final volume of 100 μl. The expression of IL-12p40 mRNA and IL-12p70 protein was induced by the addition of 1 μg/ml *E. coli *LPS Serotype 0111:B4 (Sigma) and 10 ng/ml rIFN-γ (Pharmingen, San Diego, CA.), for the last 12 h of each culture post siRNA transfection.

### RNA isolation and semi-quantitative RT-PCR (sqRT-PCR)

Total cellular RNA was isolated from J774A.1 cells from *in vitro *experiments with TRIZOL Reagent (Gibco-Invitrogen) following the manufacturer's protocol and quantified by spectrophotometry. RNA was reverse transcribed, and 100 ng of the complementary DNA product amplified by PCR as previously described [51] using 60 ng of gene specific upstream and downstream primers. Murine β-actin product was used to normalize RNA samples. PCR conditions included a pre-incubation at 95°C for 5 min followed by 35 amplification cycles (95°C, 1 min; 1 min at annealing temperature; 2 min at 72°C, and a final 10 min at 72°C). Upstream and downstream primers for IL-12p40 were specifically designed to flank the IL-12p40 siRNA target region; sense, 5'-AAACAGTGAACCTCACCTGTGACAC-3' ; antisense, 5'-TTCATCAGCAAGTTCTTGGGCG-3'. PCR products were visualized by UV illuminated agarose gel electrophoresis.

### *In vivo *siRNA interference

Control mice (BALB/c & IL12p40^-/-^) received 200 μl Opti-mem intra-peritoneal (i.p.) containing oligofectamine alone. In addition LPS positive control mice received 1 μg *E. coli *LPS. For each experimental administration, 10 μl siRNA duplexes (IL-12p40 or controls at equimolar concentration) were premixed with 40 μl of Opti-mem. Separately, 6 μl of oligofectamine was mixed with 24 μl of Opti-mem and incubated at room temperature for 15 min. These solutions were mixed at room temperature for 40 min. For co-injection experiments, these were combined with LPS (1 μg/mouse) and formulated as above. For therapeutic silencing, mice received 1 μg LPS, in the absence of siRNA duplexes, 1 h prior to administration of siRNA (IL-12p40 or controls) as above. At various time points, blood serum, peritoneal cells or lavage fluid were sampled for further analysis.

### Peritoneal Lavage & Serum preparation

Peritoneal cells were harvested by washing the peritoneal cavity with 1 ml of sterile PBS. This was centrifuged for 5 min at 400 g, lavage supernatant was removed for analysis and cells analysed by flow cytometry. Serum was prepared by cardiac puncture. Sera and lavage supernatants were assayed without delay or storage.

### Flow Cytometry

Phenotypic analysis of siRNA-transfected cells was performed using a FACScalibur™ with associated Cellquest™ software (Becton Dickinson, San Jose, CA). Forward and side scatter were measured from peritoneal lavage preparations at 12, 24 and 48 h in response to simultaneous delivery of IL-12p40 siRNA and LPS, and at 24 h for those mice receiving therapeutic IL-12p40 siRNA post LPS administration. Cell surface marker analysis of CD11b, CD14, CD40, CD80, CD86, F4/80 and MHC class II by J774A.1 cells was performed as previously described [52], control samples included cells incubated with isotype matched, directly conjugated, control antibodies as appropriate.

### Analysis of cytokine production

Cytokine production from *in vitro *experiments was assayed using commercially available immunoassays for mouse IL-12p70, IFN-γ, IFN-β, IL-10, and IL-4 (Pharmingen). Mouse IL-12p40 in blood serum and peritoneal lavage fluid was assayed using murine IL-12p40 ELISA (R&D systems, Abingdon, UK) according to the manufacturer's instructions.

### Statistical analysis

One-way ANOVA was used to determine significance of cytokine production between groups; post test analyses were not performed. The student t-Test was used to determine the significance of different fluorescent intensities obtained by flow cytometry.

## Results

### IL-12 p40 siRNA knocks down IL-12 expression *in vitro*

**Figure 1:**
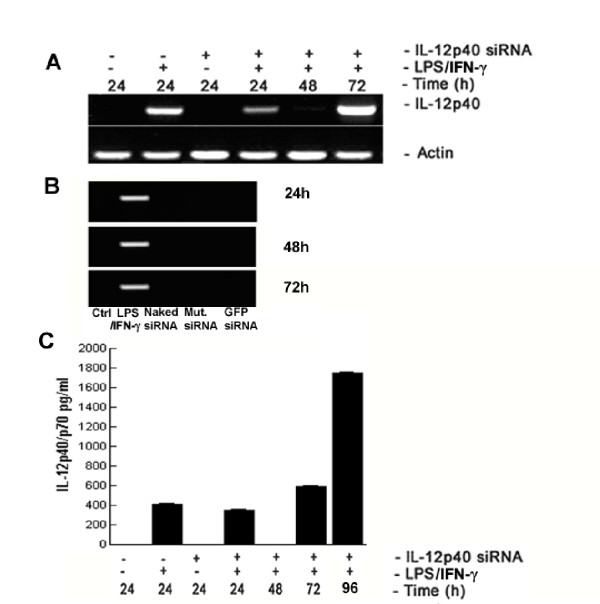
siRNA interference of IL-12 transcription and translation *in vitro*. J744A.1 cells were transfected with IL-12p40 siRNA for different periods (24–72 h). For the last 12 h of culture, cells were stimulated with LPS/IFN-γ or with PBS (-LPS, hereafter termed unstimulated). Expression of IL-12p40 (A) was determined by sqRT-PCR. Samples were normalized for β-actin expression (lower panel A). Control siRNA transfections included naked siRNA for IL-12p40, mutant siRNA for IL-12p40 and GFP (B). IL-12p70 protein expression was determined by ELISA (C). Data are representative of at least four independent experiments; IL-12p70 protein concentration is expressed as the mean (+/-SEM) from triplicate cultures (n = 3) on each occasion.

**Figure 2:**
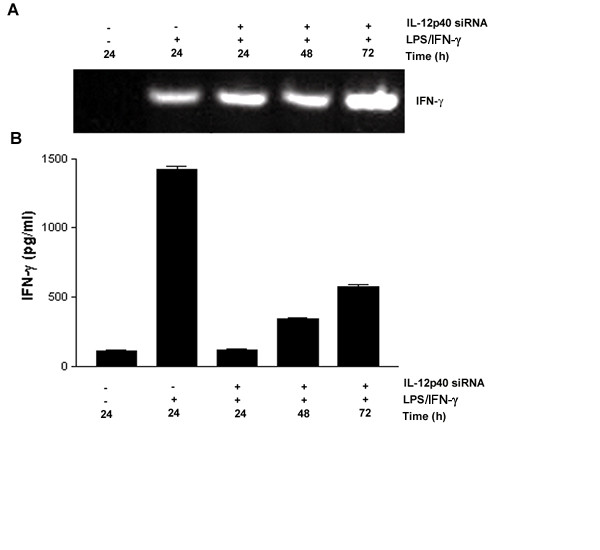
Silencing IL-12p40 influences IFN-γ protein expression. The influence of silencing IL-12p40 on the expression of IFN-γ was determined using the conditions described in Fig. 1, by sqRT-PCR for IFN-γ mRNA (A) or by ELISA for IFN-γ protein present in culture supernatant (B). Data are representative of at least four independent experiments; IFN-γ protein concentration is expressed as the mean (+/-SEM) from triplicate cultures (n = 3) on each occasion. Levels of IFN-γ are significantly reduced in the presence of IL-12p40 siRNA for 24, 48, and 72 h (p < 0.0001) when compared to stimulated non-silenced cultures.

To investigate silencing of cytokine gene expression *in vitro, *the murine macrophage-like cell line J774A.1 was transiently transfected with siRNA targeting IL-12p40 for the time points shown in Fig. 1 (24, 48, 72 h). These cells were stimulated for the final 12 h of each experiment, with LPS and IFN-γ (LPS/IFN-γ), a protocol that induces IL-12p70 [30]. Transfection with IL-12p40 siRNA resulted in a significant suppression of p40 mRNA and a consequent loss of detectable IL-12p70 in cell culture supernatant (Fig. 1A,1B,1C). A reduction in IL-12p40 mRNA was observed at 24 h, but silencing was more pronounced at 48 h. Transfection for 72 h with IL-12p40 siRNA was inferior to either 24 or 48 h, as IL-12p40 mRNA expression and IL-12p70 protein synthesis began to recover by this time (Fig. 1A &1C). Thus siRNA silencing was transient in this system. Control siRNA transfections included siRNA for IL-12p40 without transfection agent (naked siRNA), siRNA for IL-12p40 where the 10^th ^and 11^th ^bases were reversed (mutant siRNA), and siRNA targeting GFP, a protein that does not naturally occur in J774A.1 cells. These control siRNAs did not induce IL-12p40 mRNA expression (Fig. 1B). Our results show sequence-specific siRNA mediated inhibition of IL-12p40 mRNA synthesis *in vitro *at 48 h post siRNA incubation (Fig. 1A). ELISA confirmed the siRNA mediated silencing of IL-12p70 protein expression (Fig. 1C), reflecting the significant inhibition of IL-12p40 mRNA synthesis (p < 0.001, compared to LPS/IFN-γ group). Supernatants from unstimulated cells, or cells incubated with control siRNAs, showed no IL-12p70 protein production. Suppression of IL-12p70 was transient, with levels recovering at the remaining time points. mRNA expression profiling for the inflammatory cytokines IFN-β, IL-12p35, IL-23p19, IL-6, IL-10 and IFN-γ in IL-12p40 or control silenced cells, showed no non-specific siRNA silencing at the doses employed (Table 1). Control siRNA delivered by the same protocol did not induce mRNA for IFN-β, IL-12p35, IL-23p19, IL-6, IL-10 and IFN-γ. Likewise, cells transfected with IL-12p40 siRNA showed no modulation of the protein levels of IL-4, IL-5, IL-6, IL-10, and TNF-α (results not shown). One cytokine did not follow this pattern. Although IL-12p40 siRNA transfection of stimulated macrophages did not result in a detectable reduction of IFN-γ mRNA (Table 1 and Fig. 2A), a reduction of detectable IFN-γ protein was observed (Fig. 2B). This discrepancy between IFN-γ mRNA and protein detection may reflect the role of IL-12p40 in post-transcriptional regulation of IFN-γ secretion [31] and is consistent with the timing of IFN-γ protein synthesis and secretion previously observed in LPS challenged IL-12p40^-/- ^mice *in vivo *[32].

**Table 1:** IL-12p40 siRNA specifically silences mRNA for IL-12p40 and not other cytokines.

Target	Silencing by treatment^a^		
LPS/IFN-γ:	-	+	+
SiRNA:	IL-12p40	Mut.siRNA	IL-12p40

β-Actin	-	-	-
IL-12p40	-	-	+
IL-12p35	-	-	-
IL-23p19	-	-	-
IFN-γ	-	-	-
IFN-β	-	-	-
TNF-α	-	-	-
IL-4	-	-	-
IL-5	-	-	-
IL-6	-	-	-
IL-10	-	-	-

^a^J774 cells were incubated with or without LPS/IFN-γ and specific or control siRNA as described in the materials and methods section. mRNA for different targets were detected by sqRT-PCR. In this table silencing (+) is defined as the loss of a visible band from stimulated cultures; the - symbol indicates either no loss of a visible band from stimulated (siRNA + LPS/IFN-γ) cultures, or no visible alteration (induction or loss) in unstimulated (siRNA – LPS/IFN-γ) cultures. All results represent at least two experiments performed in triplicate.

### Silencing IL-12p40 reduces LPS/IFN-γ driven macrophage activation *in vitro*

To determine whether silencing IL-12p40 had broader effects on macrophages, the expression of the activation/co-stimulatory markers CD40, CD80, CD86, and MHC class II was examined following simultaneous exposure of J774 cells to LPS/IFN-γ and either control or IL-12p40-specific siRNA. Expression of CD14, a component of the LPS recognition machinery was also examined. LPS/IFN-γ stimulation alone (24 h) resulted in increased CD40, CD86 and MHC class II expression (Table 2), but had no effect on CD80 or CD14 as expected. IL-12p40-specific siRNA did not activate macrophages in the absence of LPS/IFN-γ (Table 2). In the presence of LPS/IFN-γ, siRNA targeting IL-12p40 prevented increased expression of CD40, and CD86, suggesting that silencing IL-12 interfered with macrophage activation. The expression of CD80, CD14 and MHC class II were not affected (Table 2). In contrast, Mut.siRNA did not prevent CD86 upregulation when cells were stimulated with LPS/IFN-γ but rather resulted in increased expression, suggesting that this sequence may contribute to macrophage activation not seen with IL-12p40 specific siRNA. The expression of the macrophage phenotypic markers CD11b and F4/80 were unchanged in all experiments, no significant difference was seen in levels of apoptosis between groups (data not shown).

**Table 2:** LPS/IFN-γ driven macrophage expression of CD40 and CD86 is modulated by IL-12p40 siRNA.

Treatment		Mean Fluorescent Intensity (+/-SEM)				
					
LPS/IFN-γ	siRNA	CD40	CD86	CD80	CD14	MHCII
-	-	22 (5)	9 (4)	34 (8)	44 (11)	19 (8)
-	IL-12p40	16 (9)	14 (1)	29 (2)	49 (4)	18 (8)
+	-	290 (27)	41(5)	102 (21)	86 (11)	46 (5)
+	IL-12p40	94 (17)*	7 (1)*	66 (13)	47 (9)	46 (5)
+	Mut.siRNA	241 (18)	72 (6)	102 (4)	86 (16)	43 (5)

Data are the mean ± SEM to nearest whole number of MFI of 3 independent experiments, each performed in triplicate (n = 3), * statistical significance (α = 0.05) compared to cells stimulated with LPS/IFN-γ in the absence of IL-12p40 siRNA.

### siRNA targeting IL-12p40 specifically reduces LPS driven inflammation *in vivo*

**Figure 3:**
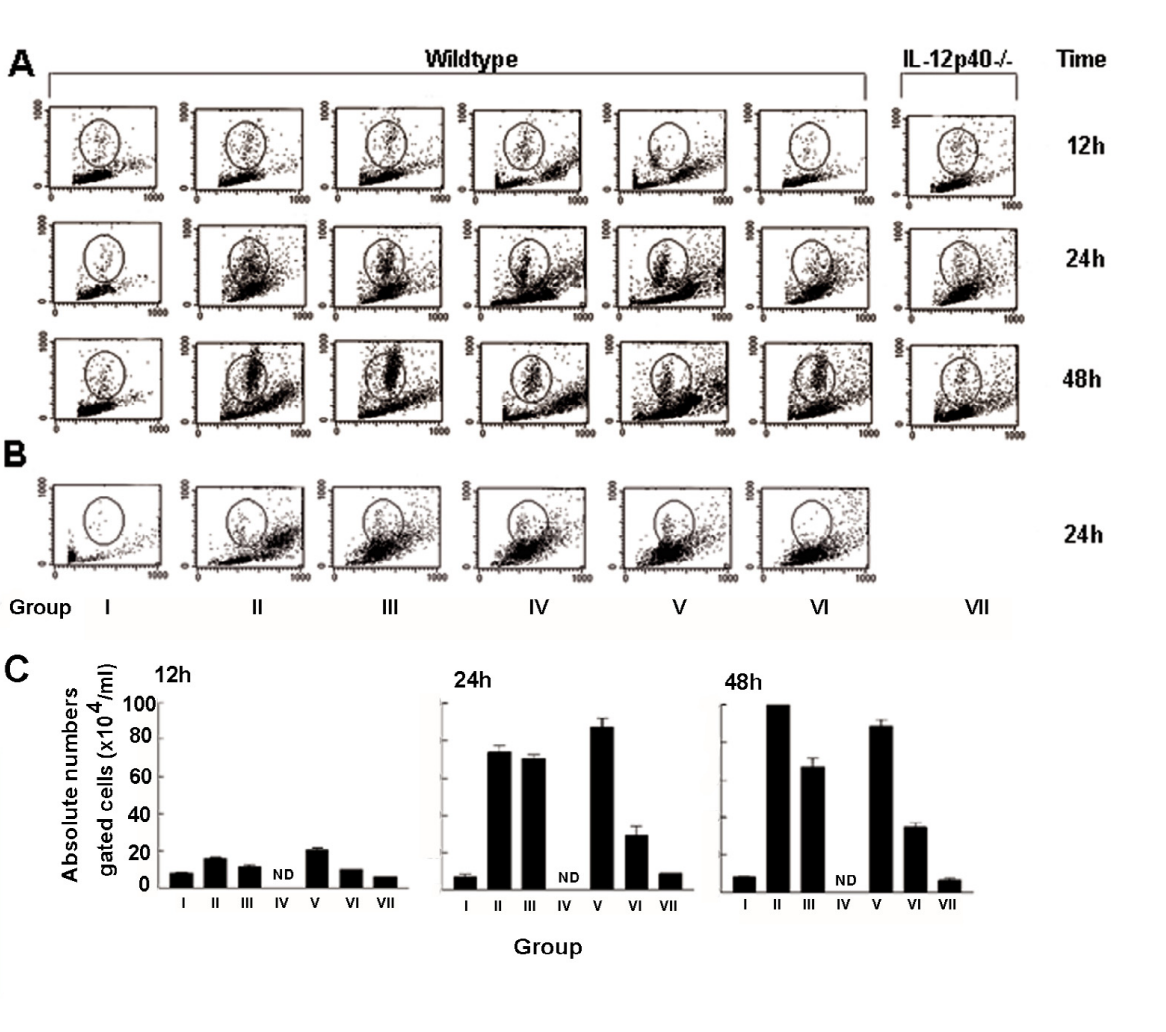
Silencing of IL-12 by siRNA interferes with the inflammatory response *in vivo*. siRNA was delivered with LPS (A) or therapeutically 1 h post LPS stimulation (B). Mice received transfection reagents only (no siRNA) (group I), LPS alone (group II) or were co-injected with LPS and control siRNAs (group III-V, As.siRNA, GFPsiRNA, and Mut.siRNA respectively) or LPS and IL-12p40 specific siRNA (group VI). Inflammation was characterized by flow cytometry of peritoneal lavage at 12, 24 and 48 h. The typical inflammatory cell response in the peritoneal cavity is shown in the enclosed region. Control IL-12p40^-/-^mice showed the characteristic germ-line knockout response to LPS throughout the experiment (group VII). IL-12p40 siRNA was also delivered therapeutically (B) 1 h post LPS challenge, and inflammation measured at 24 h. Mice receiving control siRNAs (groups III-V) displayed a similar inflammatory response to mice receiving LPS insult alone (group II). Mice receiving IL-12p40 siRNA (group VI) displayed a reduced number of activated phagocytic cells at the same time point (enclosed region). Data are representative of at least three independent experiments (Groups I-VI) or two experiments (Group VII). In each experiment, n = at least 4 mice on each occasion. The absolute numbers of cells present in lavage fluid, represented by the enclosed region in A, are illustrated (C). Data in bar charts represent the mean number of cells (+/-SEM)/ml lavage fluid from four mice at the time points indicated.

We investigated the possibility of silencing IL-12 by RNA interference *in vivo*, using a well-established murine model of LPS driven peritoneal inflammation [33,34]. The delivery of LPS i.p. resulted in increased activated phagocytic cells detectable at 12, 24 and 48 h by lavage, compared to controls (Fig. 3A groups I & II). This effect was greatly reduced in IL-12p40^-/- ^mice. Simultaneous delivery of a control irrelevant siRNA (GFPsiRNA) or a mutant IL-12p40 siRNA (Mut.siRNA) duplex containing two mismatches to the IL-12p40 specific sequence had no influence on LPS driven inflammation. Likewise, a control siRNA that was the antisense of the functional duplex (As.siRNA) did not result in a significant reduction in the level of activated phagocytic cells. However, delivery of IL-12p40 siRNA dramatically reduced the levels of inflammation (Fig. 3A) at 12, 24 and 48 h. Delivery of encapsulated siRNA did not result in increased in cell death of peritoneal cells (apoptosis or necrosis) compared to controls at the time points selected (data not shown).

Control wildtype and IL-12p40^-/- ^mice showed no inflammatory response to siRNA transfection reagents alone (Fig. 3). LPS challenged wildtype mice, and mice co-challenged with control siRNAs displayed a typical inflammatory cell response in the peritoneal cavity with increased numbers of activated phagocytic cells, at 24 h (19.8%, 16.8 %, 22.01%) and 48 h (22.53%, 17.95% 16.64%) compared to control unchallenged mice (2.17% and 3.46%). Similar results were observed in mice co-challenged with LPS and As.siRNA at 24 and 48 h (18.9% and 23.64% respectively). However, mice co-administered LPS and specific IL-12p40 siRNA displayed reduced numbers of activated cells (6.3%), mirroring the reduced inflammatory response seen in LPS challenged IL-12p40^-/- ^mice (7.3%) at 24 h. However, modulation of the inflammatory response in the LPS-IL-12p40 siRNA challenged mice was not permanent. An increase in activated phagocytic cells (11.10%) was seen at 48 h, although levels were still lower than the LPS challenged BALB/c mice (22.53%), (Fig. 3A &3C). Thus, siRNA-mediated silencing of IL-12p40 mRNA in this model has a significant but non-permanent effect on the ability to mediate a response to LPS challenge *in vivo*.

### siRNA targeting of the proinflammatory cytokine IL-12p40 can be used as a therapeutic intervention against inflammation driven by microbial products

In order to explore the potential use of siRNA in a more therapeutic context and based on the findings above, we delivered IL-12p40 siRNA by direct injection into the peritoneal cavity, 1 h post LPS challenge. Administration of IL-12p40 siRNA post LPS challenge (Fig. 3B I-VI) resulted in a decrease in the number of activated phagocytic cells, (4.22%) at 24 h, when compared to mice receiving LPS only (16.64%), control siRNAs (Mut.siRNA and GFPsiRNA) or As.siRNA, (25.03%, 17.07% and 12.63% respectively). These data demonstrate that IL-12p40 siRNA can be used therapeutically to specifically silence a cytokine-driven inflammatory response *in vivo*, if delivered at a suitable moment.

**Figure 4:**
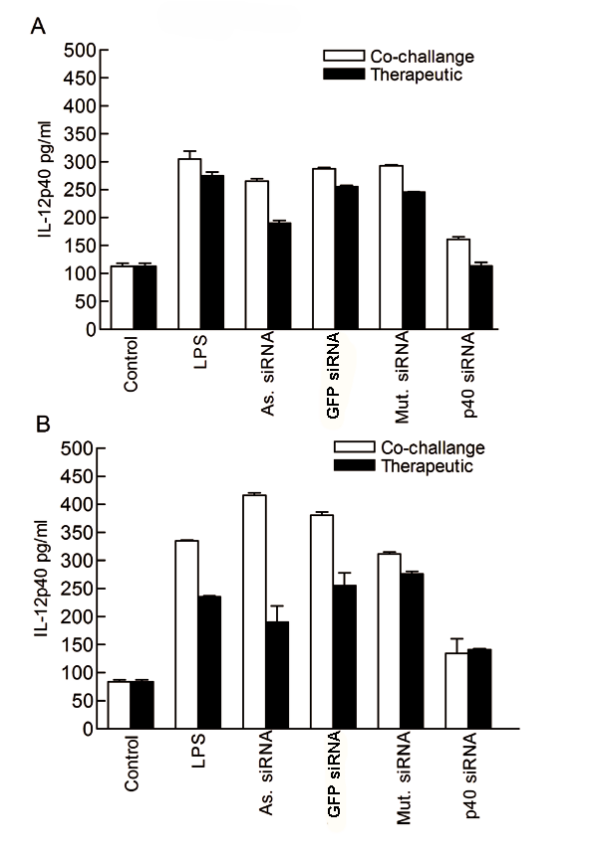
siRNA silencing IL-12p40 reduces local and systemic levels of IL-12p40 protein. LPS delivery to the peritoneal cavity with simultaneous (white bars), or therapeutic administration (black bars) of IL-12p40 specific siRNA reduced the appearance of IL-12p40 protein in serum (A) and peritoneal lavage (B) as measured by IL-12p40 specific ELISA. Serum and lavage were sampled at 6 h post LPS challenge. IL-12p40 observed in serum or lavage from LPS challenged mice or mice co-challenged with LPS and either GFPsiRNA or Mut.siRNA was significantly greater than control (p < 0.05, in both cases). IL-12p40 siRNA when delivered simultaneously or therapeutically significantly reduced IL-12p40 protein production compared to LPS challenge alone (p < 0.05, in each case). Data are representative of three independent experiments where n = 4 on each occasion; IL-12p40 protein concentration is expressed as the mean (+/-SEM) from triplicate samples.

In parallel experiments, the local and systemic effects of siRNA mediated silencing were assessed. IL-12p40 siRNA co-delivered with LPS or administered post LPS insult, had both local and systemic anti-inflammatory effects (Fig. 4). Control BALB/c mice given siRNA transfection reagents alone, showed low levels of IL-12p40 protein expression in blood serum and peritoneal lavage samples (103 pg/ml and 75 pg/ml respectively). However, mice challenged with LPS, or co-challenged with LPS and control siRNAs (GFPsiRNA, Mut.siRNA)(Fig. 4) showed significant increases in IL-12p40 protein detected in both serum and lavage compared to control (p < 0.05). Delivery of As.siRNA did result in reduced serum IL-12p40 protein but only when administered therapeutically (Fig. 4B). Strikingly, delivery of IL-12p40 siRNA delivered simultaneous to, or 1 h post LPS administration, resulted in a significant reduction in the levels of IL-12p40 protein detected in all serum and peritoneal lavage samples compared to LPS alone (p < 0.05, in each case) (Fig. 4). Delivery of negative control siRNAs showed no such reduction. Our findings demonstrate that well designed sequence specific siRNA can provide a significant therapeutic effect and elicit local and systemic protection against inflammation.

## Discussion

The ability to efficiently deliver small interfering RNA to modulate gene expression *in vivo *may provide new therapeutic approaches to currently intractable diseases. We initially demonstrate that *in vitro *IL-12p40 siRNA specifically silenced its mRNA homologue leading to transient silencing of IL-12p40 protein and consequent knockdown of IL-12p70 expression. This approach did not target other proinflammatory cytokines (IL-6, IL-23, IL-10, TNF-α), for RNA-induced gene silencing, nor did control siRNA induce these cytokines or type I interferon at the concentrations employed. Furthermore, we demonstrate that this approach can be extended *in vivo *by showing that silencing of IL-12p40 results in the non-permanent suppression of IL-12 in a murine model of peritoneal inflammation. Such silencing is evident in the reduced levels of IL-12 detectable locally in peritoneal lavage and systemically in blood serum. Finally, we show that siRNA can be used therapeutically after the initiation of an inflammatory response to knockdown IL-12 expression and to reduce the observed inflammatory infiltrate seen in this model.

IL-12 is a key factor in the early inflammatory response and in the subsequent development of type 1 responses [35]. A variety of signals can stimulate macrophages resulting in increased surface expression of CD40 and the B7 family member CD86 as well as activation of the cell's antimicrobial machinery [20,36]. Here, we show that silencing of IL-12p40 interferes with endotoxin mediated activation as measured by CD40 and CD86 expression, similar to that seen in IL-12p40^-/- ^mice in which macrophages adopt the so-called M2 profile [37]. Taken together, these data support the hypothesis that IL-12p40 has a central role in driving macrophage polarization, and regulating the intrinsic ability to respond to immunological insult [30,37]. The polarization of CD4^+ ^T-cell cytokine production towards type 1 or type 2 responses following immunological insult is controlled by a number of factors, including the nature of the immunogen, route of immunization, the APC and the regulatory cytokine milieu at the site of T-cell stimulation [38,39]. IL-12 induces the secretion of IFN-γ by NK and CD4^+ ^T-cells, promoting the differentiation and development of Th1 cells from Th0 precursors [40,41]. Th1 cells play an important role in the resolution of infections with intracellular organisms, IL-12 influences the course of bacterial, viral, and parasitic infections by altering the balance of Th1 and Th2 cells in favour of IFN-γ production [42,43]. The ability to transiently silence IL-12 may therefore be a useful research tool to dissect the development of polarized immune responses in a variety of infectious diseases.

Although IL-12p40 as a component of IL-12p70 is known to have a direct role in macrophage activation [36,44], it has recently become clear that IL-12p40 has a role independent of the heterodimer [24]. IL-12p40 acts as an antagonist of IL-12p70 function [23], but also has direct effector function [25,45]. In particular IL-12p40 plays a role in macrophage, but not NK or T-cell recruitment and chemotaxis to inflammatory sites [25,45]. The silencing of IL-12p40, and subsequent reduced inflammation seen *in vivo *during the present study supports a broader role for IL-12p40 in macrophage recruitment to sites of inflammation induced by microbial stimuli. Silencing IL-12p40 *in vitro *did not result in non-specific silencing of IL-12p35, IL-23p19, IL-10, TNF-α, IL-6 or IFN-γ mRNA. However, silencing of IL-12p40 by siRNA did result in a reduction of IFN-γ production detected by ELISA. Regulation of IFN-γ production by macrophages has not been extensively studied, however it has been shown that in some cell types IL-12 promotes nuclear localization of IFN-γ mRNA and exerts post-transcriptional control over IFN-γ secretion [31]. Our observations are consistent with this finding and suggest that IL-12 may exercise post-transcriptional control on IFN-γ protein production in macrophages.

Non-specific immune stimulation is an undesirable side effect of antisense oligonucleotides and vector based expression approaches *in vivo *[8,46]. Recently Sledz et al., (2003) have found that under some conditions transfection of siRNA results in IFN-mediated activation of the JAK-STAT pathway and global upregulation of IFN-stimulated genes. To demonstrate specificity of gene suppression, and non-activation of the IFN immune response in our study, three siRNA duplexes were designed according to Semizarov et al. [46]. We employed three different control siRNAs; a mutant IL-12p40 siRNA (Mut.siRNA) with two point mutations at the 10^th ^and 11^th ^nucleotide of the IL-12p40 siRNA duplex, an irrelevant siRNA duplex (GFPsiRNA) [26] and also the antisense of the siRNA duplex (As.siRNA). At the concentrations employed in this study we saw no non-specific silencing from control siRNA and notably no induction of IFN-β.

The ability to silence an inflammatory mediator *in vivo *has implications for the application of siRNA approaches in inflammatory diseases such as sepsis, acute respiratory distress syndrome, and T-cell mediated autoimmune diseases where the transient suppression of inflammatory gene expression would be likely to prove beneficial [47]. We demonstrate that delivery of liposome-encapsulated siRNA targeting IL-12p40 to the murine peritoneal cavity can moderate an inflammatory stimulus *in vivo*. To date there have been very few demonstrations of siRNA efficacy *in vivo*. It has been shown that intravenous injection of Fas siRNA specifically reduced Fas mRNA levels and expression of Fas protein in mouse hepatocytes [6]. More recently Sorensen et al, reported siRNA mediated TNF-α protein ablation *in vivo *[48]. Using a similar delivery technique, our study greatly expands the use of siRNA as a pharmaceutical tool for drug discovery by demonstrating that i.p. delivery inhibits endogenous gene expression affecting detectable cytokine levels both locally and systemically, resulting in altered cell activation and maturation during inflammatory insult. This supports the findings of Song et al, who showed that treatment with Fas siRNA 2 days prior to mitogen challenge abrogated hepatocyte necrosis and inflammatory infiltration resulting in reduced serum concentrations of transaminases [6]. We investigated whether IL-12p40 specific siRNA could be used therapeutically after endotoxin challenge to reduce both the local and systemic inflammatory response. Our results show delivery of IL-12p40 siRNA provides local and systemic anti-inflammatory effects on IL-12p40 protein levels. Thus, the delivery of siRNA can be used to elicit specific, non-permanent, inhibition of endogenous protein expression after exposure to inflammatory insult.

The simplicity of this approach provides a rapid means to elucidate novel druggable targets in formerly intractable inflammatory and immune-mediated diseases. Importantly, the transient and specific silencing of protein products may prove advantageous. Permanent gene silencing through the use of plasmid DNA reporter systems [2,26] retroviral [27] or lentiviral expression vectors [29] have a number of disadvantages. Permanent silencing of immune mediators may leave the host susceptible to subsequent infection, or set up potentially pathological hyper-responsiveness. Furthermore, there are unresolved safety issues relating to gene integration and host cell transformation that render these approaches less acceptable for use in children. The use of non-heritable siRNA delivered by liposomes circumvents these problems and opens the way for exploring the use of siRNA in humans to treat disease.

Our findings show that synthetic siRNA molecules delivered by intra-peritoneal injection are not affected by serum derived exonuclease activities [49] and do not require structural variations or stabilizing modifications [50] in order to have an efficient local and systemic effect on the target gene. This approach is effective, non-permanent, technically simple, and avoids some of the side effects of other delivery and gene silencing approaches.

## Conclusions

We have demonstrated *in vitro *that IL-12p40 siRNA specifically silenced its mRNA homologue leading to transient silencing of IL-12p40 and consequent knockdown of IL-12p70 expression. At the doses employed, this was specific and did not result in detectable induction of type I interferons. Silencing of IL-12p40 by siRNA did result in a reduction of IFN-γ production detected by ELISA. These findings were extended *in vivo*. Silencing of IL-12p40 results in the non-permanent suppression of IL-12 in a murine model of peritoneal inflammation. We show that siRNA can be used therapeutically after the initiation of an inflammatory response to silence IL-12 expression and observe a reduction in peritoneal inflammatory infiltrate.

## Competing Interests

The author(s) declare that they have no competing interests.

## Author's Contributions

BPM directed the study and experimental design. DGC and MAF designed siRNA molecules and primers, and executed the experimental procedures. SMT participated in the experimental design. All authors read and approved the final manuscript.
